# Turkey hen sperm storage tubule transcriptome response to artificial insemination and the presence of semen

**DOI:** 10.3389/fphys.2023.1305168

**Published:** 2024-01-08

**Authors:** Kristen Brady, Katina Krasnec, Charlene Hanlon, Julie A. Long

**Affiliations:** ^1^ Animal Biosciences and Biotechnology Laboratory, Beltsville Agricultural Research Center, Agricultural Research Service, United States Department of Agriculture, Beltsville, MD, United States; ^2^ Mouse Genetics and Gene Modification Section, Comparative Medicine Branch, National Institute of Allergy and Infectious Diseases, NIH, Bethesda, MD, United States; ^3^ Department of Poultry Science, Auburn University, Auburn, AL, United States

**Keywords:** Turkey, sperm storage tubule, fertility, artificial insemination, transcriptome

## Abstract

**Introduction:** Sperm storage within the uterovaginal junction (UVJ) of avian species occurs in specialized structures termed sperm storage tubules (SSTs) and allows for prolonged storage of semen, though the molecular mechanisms involved in semen preservation are not well understood. Little work has been done examining how function of the SSTs is impacted by insemination and by semen present in the SSTs.

**Methods:** Transcriptome analysis was performed on isolated SSTs from turkey hens receiving no insemination (control), sham-insemination, or semen-insemination at three timepoints (D1, D30, and D90 post-insemination). Bioinformatic and functional annotation analyses were performed using CLC Genomics Workbench, Database for Annotation, Visualization, and Integrated Discovery (DAVID), and Ingenuity Pathway Analysis (IPA). Pairwise comparisons and k-medoids cluster analysis were utilized to decipher differential expression profiles in the treatment groups.

**Results:** The SST transcriptome of the semen inseminated group exhibited the greatest differences within the group, with differences detectable for up to 90 days post insemination, while control and sham-inseminated groups were more similar. In the semen-inseminated samples, upregulation of pathways relating to classical and non-classical reproductive signaling, cytoskeletal remodeling, physiological parameters of the local UVJ environment, and cellular metabolism was observed. In the sham-inseminated samples, upregulation of immune pathways and non-reproductive endocrine hormones was observed.

**Discussion:** This work provides insights into the molecular level changes of the SST in response to insemination as well as to the presence of semen. Results from this study may have direct implications on fertility rates as well as potential strategies for avian semen cryopreservation protocols.

## 1 Introduction

Avian species are capable of prolonged sperm storage in the female reproductive tract due to specialized tubular glands that invaginate the mucosal folds of the uterovaginal junction (UVJ), referred to as sperm storage tubules (SST). SST differentiation immediately precedes sexual maturation, with simple columnar epithelium as the primary site responsible for prolonged fertility following a single artificial insemination or natural mating interaction (Christensen, 1981; Bakst, 1994). In avian species, duration of fertility from a single artificial insemination corresponds to the number and size of the SST present in the UVJ and varies across species. The turkey hen has been shown to possess up to 30,566 elongated SST, capable of storing sperm for up to 70 days compared to the domestic chicken, which has only up to 4,893 shorter SST, capable of storing sperm for only 21 days (Birkhead and Møller, 1992; Bakst et al., 2010). Despite stark differences in SST numbers between avian species correlated with duration of fertility, SST number does not differ between low fertility and high fertility hens within the same flock, indicating that SST functional differences are responsible for downstream differences in flock fertility rates. Further, knowledge of how SST preserve sperm function for an extended period could be exploited to improve current semen cryopreservation procedures. Semen cryopreservation has not yet proven to be economically feasible in the poultry industry, as fertility rates have been reported to decline dramatically after only 6 h of storage at 4°C (Long and Conn, 2012). The development of greater understanding of the changes occurring *in vivo* during sperm storage provides an opportunity to simulate the SST environment for the cryopreservation of semen in the poultry industry.

SST function encompasses sperm entry into the SST, sperm maintenance within the SST, and sperm release from the SST. SST selectivity acts as a significant barrier for sperm entry into the SST, with only approximately one percent of all inseminated sperm being stored. Overall, most of the sperm is expelled from the reproductive tract or triggers an immunological response, leaving only high-quality sperm to be selected for subsequent fertilization (Brillard, 1993). Within the SST, spermatozoa arrange in parallel bundles with the heads pointed at the blind end of the tubules (Bobr et al., 1964; Tingari and Lake, 1973). While in the SST, spermatozoa are maintained in a quiescent state, with a lowered rate of metabolism due to immotility (Bakst, 1987; Holm et al., 2000). SST release mechanisms have been widely investigated in regard to the timing of oviposition and ovulation, as sperm must reach the infundibulum of the oviduct shortly after follicle ovulation occurs. An initial hypothesis regarding the mechanical stimulation of release following the egg passing through the UVJ (Van Krey et al., 1967; Mero and Ogasawara, 1970) has been previously disputed by Bobr et al. (1964), who determined that spermatozoa were released around the time of follicle ovulation. This was further supported by Ito et al. (2011), who demonstrated that exogenous progesterone was able to stimulate SST sperm release. This work suggests that SST release is coordinated to occur immediately prior to the time of ovulation (Das et al., 2006).

Several studies have also investigated oviductal immunological response in virgin, sham-inseminated, and semen-inseminated layer hens. Compared to virgin hens, repeated inseminations with semen led to a reduction in fertility rates. This is suggested to be a consequence of swollen SST with a thinned epithelial layer and elevated lymphocyte populations, indicating that repeated artificial insemination (AI) will break down SST over time and reduce their functionality over the course of a breeding cycle (Das et al., 2005b). Bakst (1987) determined that virgin and sperm-inseminated hens displayed a similar number of intraepithelial lymphocytes during the later stages of the laying cycle (52 weeks), a signal that SST immune response may be inevitable regardless of sperm presence. It was proposed that this response may be triggered as the hen ages by an autoimmune reaction to an estrogen-dependent factor associated with SST (Bakst, 1987). Recently, estrogen receptor alpha (ERα) mRNA in the UVJ was observed to decline following repeat AI hens in comparison to virgin hens (Das et al., 2006). Thus, estrogen is unlikely to be the sole component of this immunological response due to changes in UVJ responsivity to this hormone in the presence of sperm. While the overall intraepithelial lymphocyte populations are elevated with age, regardless of insemination status, further investigation into lymphocytes determined that both CD4^+^ and CD8^+^ T cells were upregulated following repeated AI. T cell upregulation demonstrates a more robust cell-mediated immune response along with the capacity to stimulate macrophages, B cells, and plasma cells, that correspond to lower fertility rates (Das et al., 2005a). Interestingly, while these cell types were also present in the oviductal mucosal tissue of laying hens undergoing repeated AI (Zheng et al., 1998; 2001), elevated levels of antibody-producing plasma cells were found in the SST epithelium of infertile turkey hens (Van Krey et al., 1987). While the strong immune reaction in response to repeated AI has been identified, it remains unclear how immunity impacts SST filling and release rates, and if this response is directly linked to declining fertility.

We have previously characterized the SST transcriptome throughout the first 90 days of the production cycle following a single post-lay insemination. Through that characterization, we have identified genes involved in immune response, hormone signaling, ion homeostasis, sperm motility, and cytoskeletal reorganization that are hypothesized to play a critical role in the declining fertility rates with flock age (Brady et al., 2022). However, it remains unclear if these expression changes are the result of the presence of semen or the mechanical process of AI. To fully elucidate these differences, this study examined the transcriptomic profiles between virgin (non-inseminated), sham-inseminated, and semen-inseminated turkey hens over the course of fertility to identify the impact of sperm in comparison to mechanisms of insemination on genomic regulation and activity. The turkey industry relies exclusively on AI to produce poults used for subsequent meat production. Further investigation is necessary to differentiate SST response to the act of AI and to the presence of semen. Aside from a direct impact on flock fertility rates, a deeper understanding of SST function could also have implications on poultry semen cryopreservation protocols.

## 2 Materials and methods

### 2.1 Bird husbandry and insemination

All procedures in this study were approved by the Institutional Animal Care and Use Committee of the Beltsville Animal Research Center, United States Department of Agriculture. Commercial lines of turkey hens and toms were obtained for this study (Hendrix Genetics, Kitchener, Ontario, Canada) and housed separately. Hens were placed in individual cages to monitor egg production, and toms were kept in floor pens (*n* = 8–10 toms per pen). A ratio of 4 hens per tom was maintained throughout the study. All hens were housed under non-stimulatory photoperiods of 6L:18D to maintain the reproductive system in an immature state. At 27 weeks of age (woa), hens were photostimulated with 14L:10D to encourage the initiation of sexual maturation, inducing egg production. Toms were initially housed under 12L:12D until 28 woa, during which they were also photostimulated with 14L:10D to stimulate testicular maturation and semen production. As per the industry standard, all birds were provided *ad libitum* access to feed and water throughout the study.

Hens were randomly allocated to one of three treatments (*n* = 3 per group): no insemination (control), sham-insemination with 50 μL of Beltsville Turkey Semen Extender (Continental, Delavan, WI) only, or semen-insemination with a dose of 2.5 × 10^8^ sperm per hen + Beltsville Turkey Semen Extender in a 1:1 ratio totaling 50 μL. For the semen-inseminated group, toms were milked twice per week, simulating the conditions of the industry. This preparation began at 30 woa to familiarize the males with the abdominal message method (Burrows and Quinn, 1935) and to determine the quality of the semen through previously established screening techniques at 31 and 32 woa (Long and Kulkarni, 2004). All semen determined to be acceptable and passing the quality check were pooled at 32 woa to ensure the same pool of semen was used in all AI for the study. Two pre-lay inseminations were performed at 8 and 11 days post-photostimulation. Two weeks post-photostimulation (29 woa), a single, post-lay insemination occurred, referred to as D0.

### 2.2 UVJ tissue and SST isolation

At D1, D30, and D90, representing the days post-AI (D0), turkey hens were euthanized via pentobarbital sodium and phenytoin sodium injection (390:50 mg). Each treatment group contained 3 replicates per time point. Following euthanasia, the UVJ was dissected from the oviduct of the hen, as previously outlined by Bakst (1992). The UVJ was excised from the vagina and shell gland, and a longitudinal incision was made to display the mucosal folds known to contain SST. This region underwent microscopic analysis to identify the specific location of SST, with SST-containing regions excised. Each region was embedded in optimal cutting temperature compound (Tissue Tek, Leica Microsystems, Buffalo Grove, IL) in 25 × 20 × 5 mm cryomolds before snap freezing in liquid nitrogen and storage at −80°C. This entire procedure, from euthanasia to freezing, was conducted within 20 min. SST were isolated from the UVJ using methods previously described by Brady et al. (2022). Snap-frozen blocks were sectioned using a Lecia CM1860 cryostat at a thickness of 10 μm and positioned onto PEN membrane 2 μm slides (Leica Microsystems). Following staining with Nuclear Fast Red (Sigma-Aldrich, Inc., St. Louis, MO), individual SST structures were visualized at ×10 magnification and isolated using the Leica LMD7 (Lecia Microsystems). These structures were removed from the surrounding UVJ via laser capture microdissection techniques outlined by Brady et al. (2022) and stored at −80°C until RNA isolation. On average, 317.26 ± 11.62 SST were isolated from each slide, and the average surface area was 1,953,668.81 ± 516,597.16 μm^2^.

### 2.3 RNA isolation, quality assessment and sequencing

RNA isolation, quality assessment, and sequencing were performed as previously described (Brady et al., 2022). Isolated SST total RNA was extracted using the RNeasy Plus Micro Kit (Qiagen) according to the provided protocol as well as on-column deoxyribonuclease digestion. The RNA purity and concentration were determined using the Tapestation RNA HS Assay (Agilent, Santa Clara, CA, United States) and the Qubit RNA HS assay (Thermo Fisher Scientific, Carlsbad, CA, United States), respectively. RNA integrity number (RIN) values were determined using a Bioanalyzer 2100 (Agilent, Santa Clara, CA, United States), with an average RIN value of 8.0 ± 0.1. Libraries were constructed with the SMARTer Stranded RNA-Seq Kit (Takara Bio Inc., San Jose, CA, United States), then quantified using KAPA SYBR FAST qPCR (Roche Diagnostics, Basel, Switzerland), and quality was determined through the Tapestation RNA HS Assay (Agilent, Santa Clara, CA, United States). Sequencing was performed using the Illumina HiSeq platform according to Illumina protocols and paired end-reads of 2 × 150 bp were generated. Images were produced by the base calling pipeline using RTA 1.18.64.0 and were stored as FASTQ files.

### 2.4 Bioinformatic analysis of sequencing data

All sequencing files (Bioproject: PRJNA1022824; Biosamples: SAMN37628532- SAMN37628544, SAMN37628829- SAMN37628838, SAMN37629530- SAMN37629533) were submitted to the NIH Short Read Archive (https://www.ncbi.nlm.nih.gov/sra). Bioinformatic analysis was conducted using CLC Genomics Workbench 20.0 (Qiagen; https://digitalinsights.qiagen.com). The quality of sequence reads (raw and trimmed) was verified using FastQC (http://www.bioinformatics.babragam.ac.uk/projects/fastqc/) (Andrews, 2010) to determine sequencing read artifacts, including sites with a low-quality Phred score (<20), with reads falling below this threshold trimmed. Removal of low-quality reads was performed using the trimming tool of the CLC Genomics Workbench platform. Reads that passed quality control were aligned to the most recent *Meleagris gallopavo* reference genome (Turkey_5.1; NCBI annotation release 103: https://www.ncbi.nlm.nih.gov/datasets/genome/GCF_000146605.3/). Differentially expressed genes (DEGs) were determined using CLC Genomic Workbench, with cutoffs of a *q*-value of <0.05, an absolute fold change greater than 1.5, and an FPKM value greater than 1 (Liu and Di, 2020). DEGs were determined through pairwise comparisons between control and sham-inseminated treatment groups and between sham-inseminated and semen-inseminated treatment groups at D1, D30, and D90 timepoints. Additionally, k-medoids cluster analysis was performed at each timepoint to determine genes with expression profiles peaking in control, sham-inseminated, and semen-inseminated SST samples. Features used for clustering were restricted to genes with expression levels significantly impacted by treatment at each given timepoint. Lastly, a one-way ANOVA was performed to determine genes statistically impacted by treatment at each timepoint.

Over-represented gene ontology (GO) biological processes, cellular components, and molecular functions as well as KEGG pathways were identified using Database for Annotation, Visualization, and Integrated Discovery (DAVID), utilizing *Meleagris gallopavo* as the species to include any avian-specific mechanisms (Huang et al., 2009a; Huang et al., 2009b). DEGs were also analyzed with Ingenuity Pathway Analysis (IPA) to determine significant networks and upstream regulators (Qiagen; https://www.qiagen bioinformatics.com/products/ingenuity-pathway-analysis; Kramer et al., 2014). All three time points were examined individually using the core analysis function and then evaluated using the comparison analysis function of IPA. Generated networks with a network score of 35 or higher were considered significant. An absolute z-score greater than 2 and a *p*-value <0.05 was considered significantly activated or inhibited for the analysis of upstream gene regulators. Though very extensive, the knowledge base of IPA is currently human/mouse centric, which could exclude avian specific mechanisms occurring in SST.

### 2.5 RNA-seq gene expression validation

Reverse transcription (RT) was performed on 10 ng of all RNA-extracted samples using SuperScript III (Thermo Fisher Scientific, Waltham, MA, United States) and an anchored dT primer (Integrated DNA Technologies, Skokie, IL, United States), according to the manufacturer protocols. A negative control sample without reserve transcriptase was also prepared from pooled RNA to control for any genomic DNA contamination (no RT). RT-qPCR was conducted on 6 randomly selected DEGs to determine expression profiles. All primers (Integrated DNA Technologies, Skokie, IL, United States) were designed using primer BLAST software (NCBI, Bethesda, MD, United States) (Table 1). Primer pairs were validated as previously described (Brady et al., 2019; Brady et al., 2022). Briefly, dissociation curve analysis and gel electrophoresis were conducted for each primer pair to ensure amplification of a single PCR product of expected size and was absent from the no RT and water controls (Brady et al., 2022). Primer pairs were also validated for amplification efficiencies and sequencing of the resulting PCR product (Brady et al., 2019). Amplification was performed using the CFX Connect Real-Time PCR System (Bio-Rad, Hercules, CA, United States), using 1 μL of cDNA, 0.6 μL of each of the forward and reverse primers (final concentration in PCR reaction of 0.4 μm), 7.5 μL of 2X iTaq Universal SYBR Green Supermix (Bio-Rad, Hercules, CA), and 5.3 μL of nuclease-free water. The PCR cycling conditions were as follows: initial denaturation at 95°C for 3 min followed by 40 cycles of 95°C for 15 s, 60°C for 30 s, and 72°C for 30 s. Data were normalized to the average mean of the housekeeping genes, *TUBB3, UBB,* and *BTUBB* (Livak and Schmittgen, 2001), and the 2^−ΔΔCt^ method was used. Housekeeping genes were selected based on expression levels across the experimental groups in the RNA sequencing dataset and housekeeping gene mRNA levels resulting from RT-qPCR were confirmed to be insignificant between experimental groups through statistical analysis prior to normalization. Levels of mRNA for each gene are presented relative to the average of the control treatment group for each timepoint. SAS v9.4 (SAS Institute, Cary, NC, United States) was used to perform a Pearson’s correlation comparing the log_2_ fold change values obtained through RT-qPCR and RNA sequencing.

**TABLE 1:** Primer sequences used for confirmation of gene expression results obtained from RNA sequencing.

Gene	Forward	Reverse
*SCD*	CAA​TGC​CAC​CTG​GCT​AGT​GA	GGT​GGA​GTA​GTC​GTA​GGG​GA
*SPARC*	AAG​TGC​ACC​TTG​GAG​GGA​AC	GCG​CTT​CTC​ATT​CTC​GTG​GA
*SERPINB5*	AAG​CTA​CGT​TTT​GCC​TGG​GT	TCA​GGG​GTG​AGT​GCC​TTT​TC
*KCNMB1*	CAG​CCT​CAG​GAC​AAG​AGG​TC	GTG​AAG​AGG​AGG​CCT​TTG​GG
*SPINT4*	ATT​TCT​GCA​CGG​TCA​CTC​CC	CGC​GTT​GTA​GAA​GAA​GCG​GAT
*CYGB*	GGA​GAA​CCT​CAA​CGA​CCC​AG	CAC​GTG​GGT​GTA​GAT​GAG​GG
*UBB*	TCA​AGC​AAG​ATG​CAC​AGC​AC	TTT​CAA​CAT​ACA​GAT​CAG​CAG
*TUBB3*	CAG​TTT​TGG​GAG​GTG​ATC​AGC​GA	CCC​GCT​CTG​ACC​GAA​AAT​GA
*BTUBB*	TTG​GCC​CAC​TAT​TTC​GAC​CT	GTC​ACA​GCT​CTC​ACA​CTC​GT

## 3 Results

A total of 2.7 billion reads were obtained across the 27 samples sequenced, with an average read count of 99 ± 5.9 million reads per sample (Supplementary Figures S1A). Following quality assessment and trimming, a total of 2.4 billion reads remained, with an average read count of 88 ± 5.2 million reads per sample. On average, 86.31% ± 0.45% of reads mapped in pairs, 2.01% ± 0.13% of reads mapped in broken pairs, and 11.68% ± 0.41% of reads did not map to the turkey genome (Supplementary Figures S1B). No significant differences were identified between experimental treatments or timepoints related to reads obtained, trimmed read counts, or read mapping (*p* > 0.05).

**FIGURE 1:**
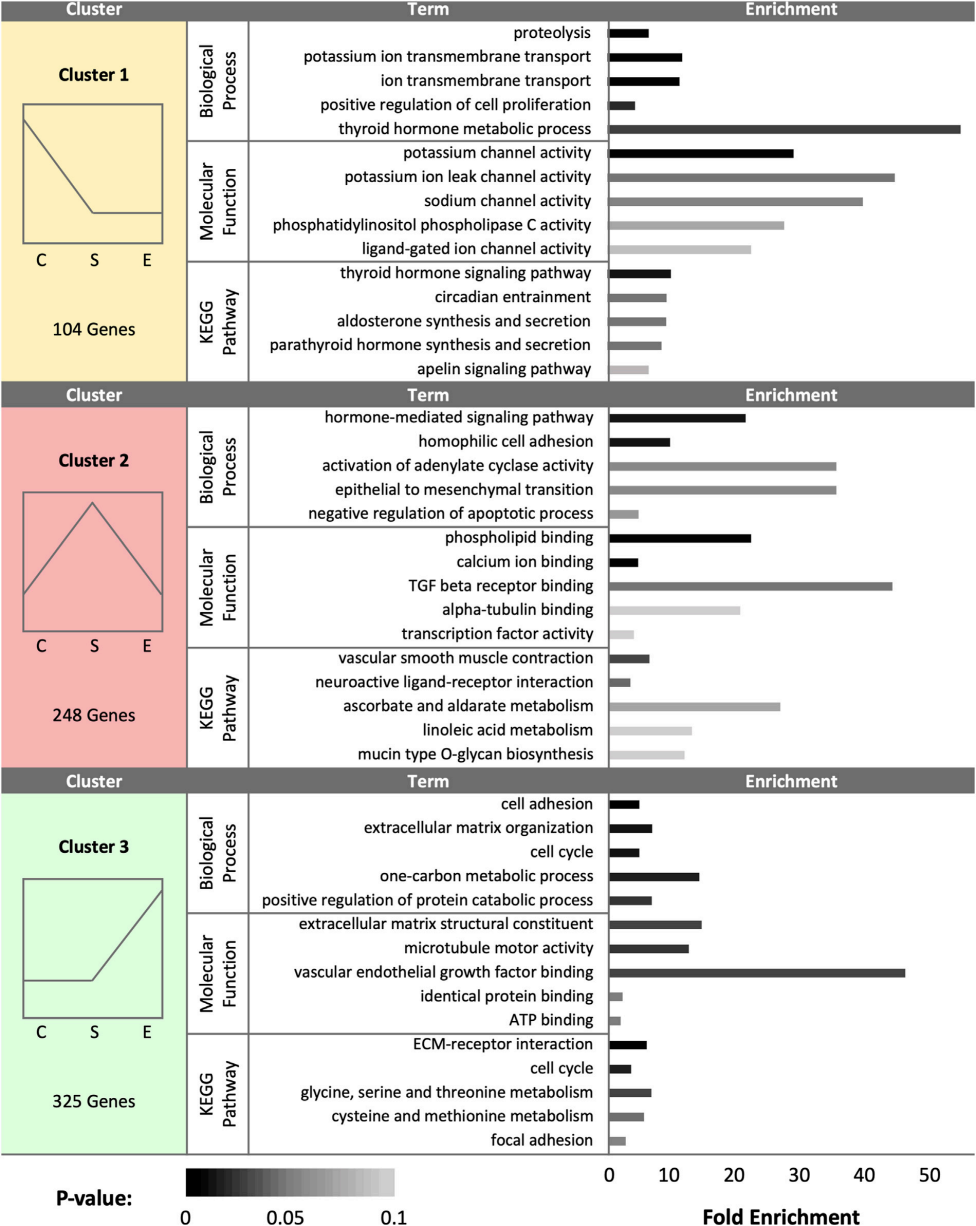
K-medoids cluster analysis of sperm storage tubules (SSTs) isolated from: control, sham-inseminated, and semen-inseminated at 1-day post-insemination (D1). The k-medoids cluster analysis tool from CLC Genomics (Qiagen, Valencia, CA) was used to identify genes exhibiting peak expression in treatment group. Genes included in the analysis were differentially expressed due to treatment (q < 0.05). Left panels show the expression profile and number of genes in each cluster (C = control, S = sham-insemination, E = semen-insemination). Right panels show enriched gene ontology (GO) term biological processes and molecular functions as well as KEGG pathway enrichment generated from Database for Annotation, Visualization, and Integrated Discovery (DAVID).

At day 1 (D1), a total of 677 DEGs were identified as statistically significant due to treatment. Cluster analysis revealed a total of 104 DEGs upregulated in control samples, 248 DEGs upregulated in sham samples, and 325 DEGs upregulated in experimental samples (Figure 1). SST isolated from control hens exhibited increased expression for genes associated with ion channel activity, thyroid/parathyroid hormone activity, cell proliferation, and proteolysis. Pathway enrichment in control hen samples was related to thyroid/parathyroid signaling, circadian entrainment, aldosterone signaling, and apelin signaling. SST isolated from sham-inseminated hens displayed upregulation of genes related to hormone response, cell adhesion, epithelial cell differentiation, and transcription factor activity. Pathway enrichment in sham-inseminated hen samples was related to muscle contraction, ligand-receptor interaction, metabolic networks, and mucin type o-glycan biosynthesis. SST isolated from semen-inseminated hens exhibited enriched gene expression linked to ECM organization, one-carbon and protein metabolism, cell division, and vascular endothelial growth factor binding. Pathway enrichment in semen-inseminated hen samples was related to amino acid metabolism, cell cycle, ECM-receptor interaction, and focal adhesion.

**FIGURE 2:**
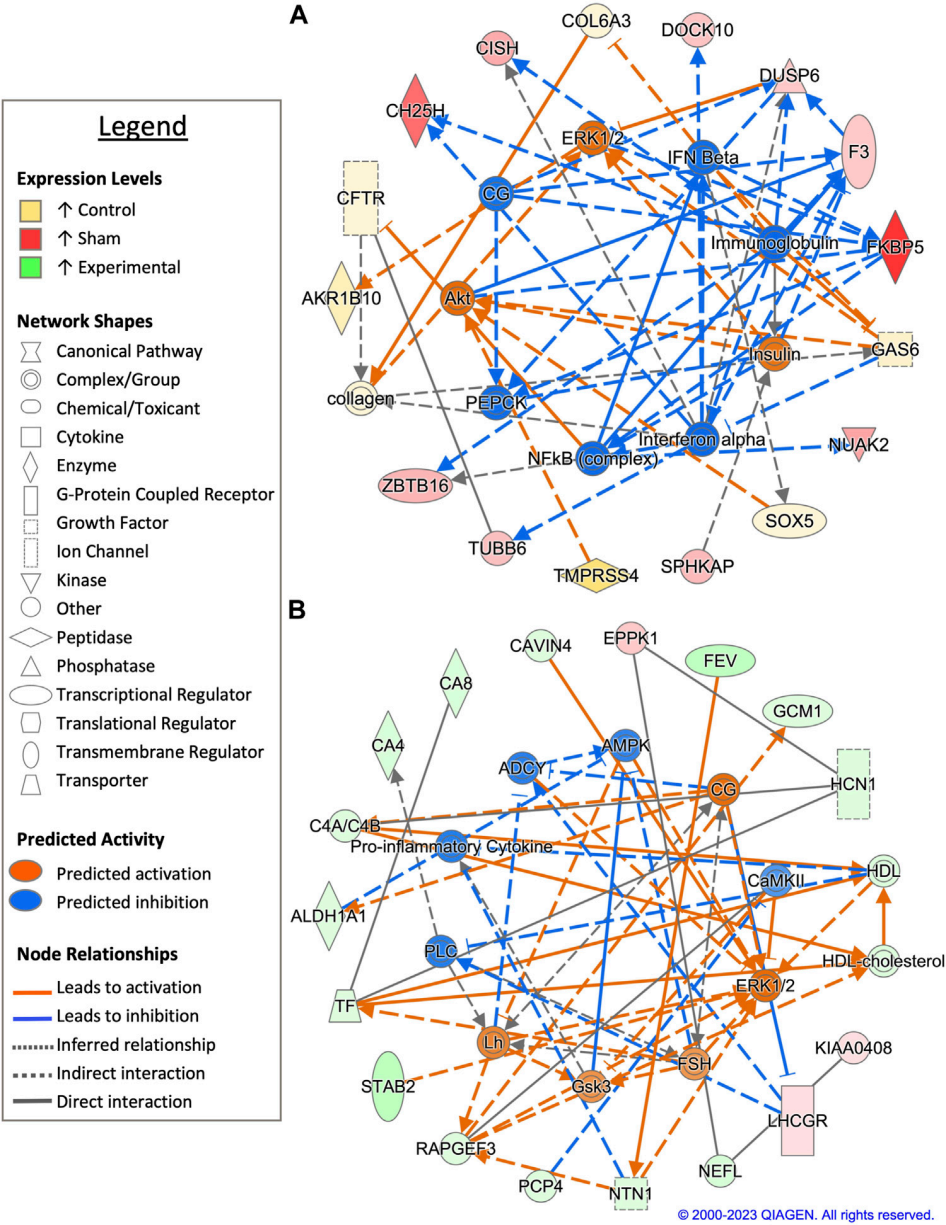
Network analysis of sperm storage tubules (SSTs) isolated at 1 day post insemination (D1). The core analysis tool from Ingenuity Pathway Analysis (IPA) (Qiagen, Valencia, CA) was used to biologically interpret differentially expressed genes (DEGs). Copyright permission from Qiagen has been obtained for use of the images presented. **(A)** The top network generated through pathway analysis of DEGs between control and sham-inseminated hens (FPKM > 1, *q*-value < 0.05, |fold change| > 1.5). **(B)** The top network generated through pathway analysis of DEGs between sham-inseminated and semen-inseminated hens (FPKM > 1, *q*-value < 0.05, |fold change| > 1.5).

Pairwise comparison between control and sham-inseminated SST at D1 generated a total of 49 DEGs, a majority of which were involved in immune response and regulation of cell proliferation (Figure 2A; Supplementary Table S1). Within the top generated network, key immune drivers, such as immunoglobulin, interferon alpha and beta, and nuclear factor kappa B (NFκB), were identified as upstream regulators, with predicted increased expression in sham-inseminated samples, leading to elevated cytokine inducible SH2 containing protein (*CISH*), FKBP prolyl isomerase 5 (*FKBP5*), and dual specificity phosphatase 6 (*DUSP6*) expression in sham-inseminated samples. These regulators are also predicted to lead to increased cell proliferation in control samples, including growth arrest specific 6 (*GAS6*) expression in control samples. In addition to the predicted regulators identified in the top generated network, upstream analysis identified eight potential regulators exhibiting significant activity with roles in immune function (*NOD2*, DUB, caspase) as well as thyroid hormone (*NKX2-1*), hypothalamo-pituitary gonadal axis (progesterone), and insulin signaling (*SPRY4*) (Supplementary Table S2). GO analysis of DEGs from the pairwise comparison between control and sham-inseminated SST at D1 showed enrichment of B cell chemotaxis, cartilage development, and chondrocyte differentiation biological processes (Supplementary Table S3).

Examination of sham-inseminated and semen-inseminated SST at D1 using a pairwise comparison identified a total of 185 DEGs (Supplementary Table S1). The top network constructed was composed of DEGs with functional roles in angiogenesis, innervation, and cytoskeletal structure (Figure 2B). The genes, stabilin 2 (*STAB2*) and rap guanine nucleotide exchange factor 3 (*RAPGEF3*), both showed increased expression in semen-inseminated samples and play roles in angiogenesis. Additionally, neurofilament light chain (*NEFL*) and netrin 1 (*NTN1*) also showed increased expression in semen-inseminated samples and play roles in innervation and cytoskeletal maintenance. Upstream regulators with classical reproductive functions, such as luteinizing hormone (LH) and follicle stimulating hormone (FSH), were predicted to be increased in semen-inseminated samples, while upstream regulators with immune functions, such as pro-inflammatory cytokine and calmodulin-dependent protein kinase II (CaMKII), were predicted to be increased in sham-inseminated samples. Upstream analysis also identified 90 potential regulators exhibiting significant activity, with one regulator, spalt like transcription factor 4 (*SALL4*), also exhibiting increased expression in semen-inseminated samples when compared to sham-inseminated samples (Supplementary Table S2). GO analysis of DEGs from the pairwise comparison between sham-inseminated and semen-inseminated SST at D1 showed enrichment of cell adhesion, angiogenesis, negative regulation of cell migration, and breakdown of glycolytic intermediates (Supplementary Table S3).

**FIGURE 3:**
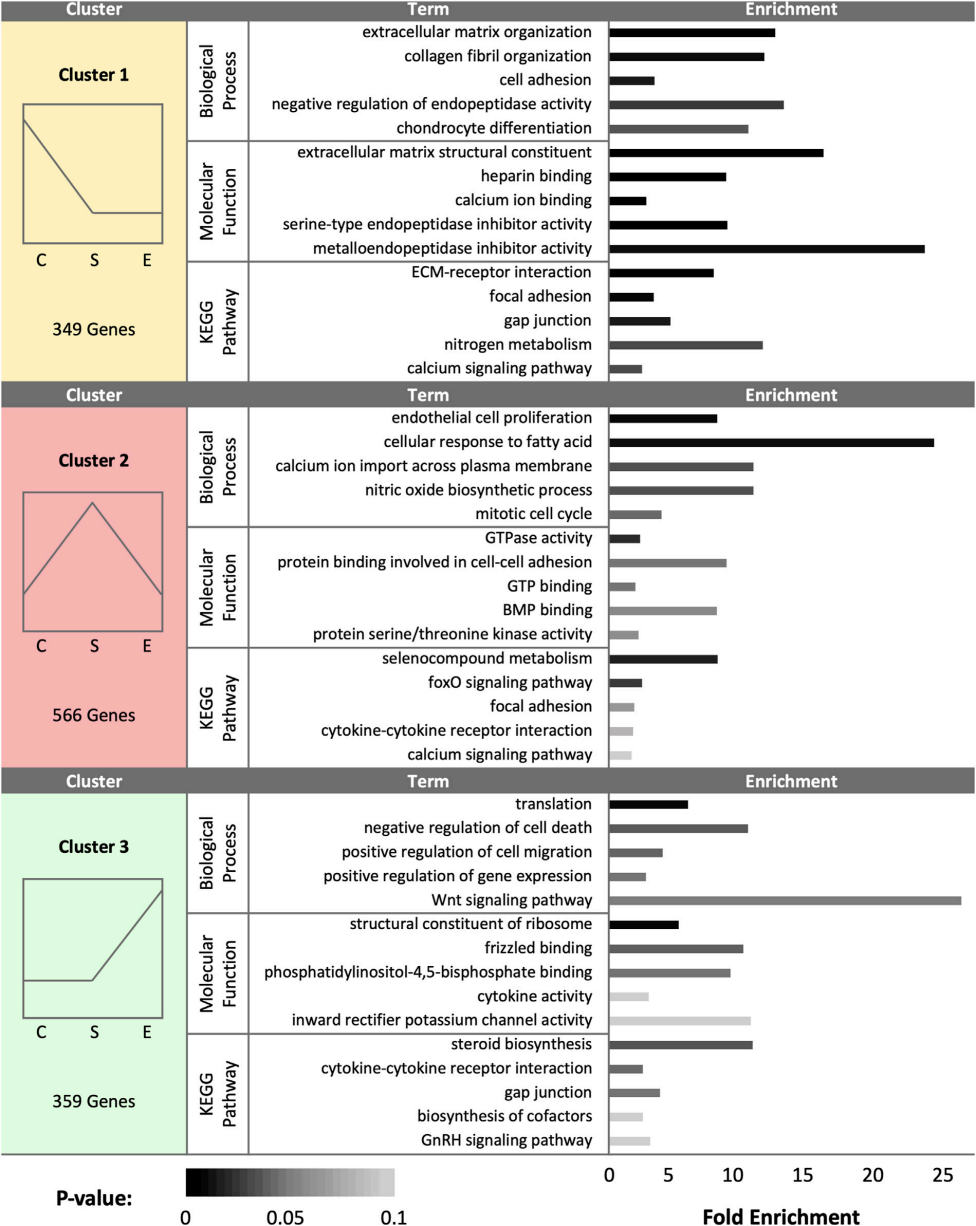
K-medoids cluster analysis of sperm storage tubules (SSTs) isolated from: control, sham-inseminated, and semen-inseminated at 30-days post-insemination (D30). The k-medoids cluster analysis tool from CLC Genomics (Qiagen, Valencia, CA) was used to identify genes exhibiting peak expression in treatment group. Genes included in the analysis were differentially expressed due to treatment (q < 0.05). Left panels show the expression profile and number of genes in each cluster (C = control, S = sham-insemination, E = semen-insemination). Right panels show enriched gene ontology (GO) term biological processes and molecular functions as well as KEGG pathway enrichment generated from Database for Annotation, Visualization, and Integrated Discovery (DAVID).

At day 30 (D30), a total of 1,274 DEGs were identified as statistically significant due to treatment group. Cluster analysis revealed a total of 349 DEGs upregulated in control samples, 566 DEGs upregulated in sham samples, and 359 DEGs upregulated in experimental samples (Figure 3). Control hen SSTs exhibited upregulation of genes associated with ECM organization, calcium and heparin binding, endopeptidase inhibitor activity, cell adhesion, and collagen production. In control hen samples, enriched pathways included ECM-receptor interaction, focal adhesion, gap junction formation, nitrogen metabolism, and calcium signaling. Sham-inseminated SSTs showed enriched gene expression linked to endothelial cell proliferation, calcium ion transport, fatty acid cellular response, nitric oxide biosynthesis, and BMP binding. In sham-inseminated hen samples, enriched pathways included cytokine-receptor interaction, selenocompound metabolism, focal adhesion, and foxO and calcium signaling. Semen-inseminated SSTs exhibited increased mRNA levels for genes related to translation, Wnt signaling, potassium channel and cytokine activity, and cell viability. In semen-inseminated hen samples, enriched pathways included steroid and cofactor biosynthesis, cytokine receptor interaction, gap junction formation, and GnRH signaling.

**FIGURE 4:**
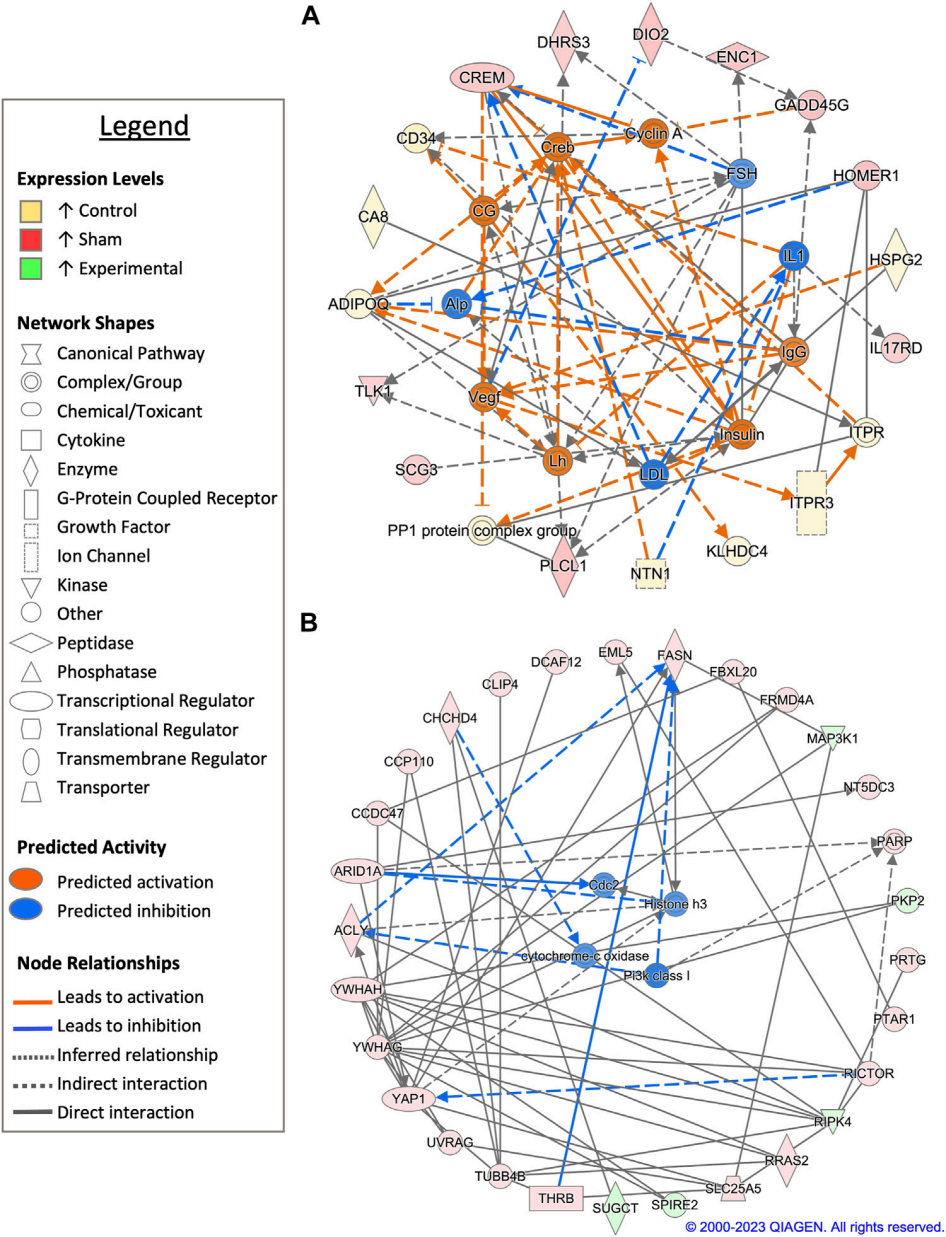
Network analysis of sperm storage tubules (SSTs) isolated at 30-days post insemination (D30). The core analysis tool from Ingenuity Pathway Analysis (IPA) (Qiagen, Valencia, CA) was used to biologically interpret differentially expressed genes (DEGs). Copyright permission from Qiagen has been obtained for use of the images presented. **(A)** The top network generated through pathway analysis of DEGs between control and sham-inseminated hens (FPKM > 1, *q*-value < 0.05, |fold change| > 1.5). **(B)** The top network generated through pathway analysis of DEGs between sham-inseminated and semen-inseminated hens (FPKM > 1, *q*-value < 0.05, |fold change| > 1.5).

Pairwise comparison between control and sham-inseminated SSTs at D30 identified a total of 59 DEGs, some of which were involved in immune response and insulin responsiveness (Figure 4A; Supplementary Table S1). Specifically, immune associated genes such as interleukin-1 (IL1) and immunoglobulin G (IgG) were predicted to have increased expression in sham-inseminated and control samples, respectively, leading to increased interleukin 17 receptor D (*IL17RD*) expression in sham-inseminated samples, while increased CD34 molecule (*CD34*) expression was identified in control samples. Additionally, insulin activity was also predicted to be increased in control samples (also seen at D1), with downstream metabolic targets, such as adiponectin, C1Q and collagen domain containing (*ADIPOQ*) and protein phosphatase 1 (*PP1*) exhibited increased expression in control samples. Upstream analysis also identified 53 potential regulators exhibiting significant activity, with regulators such as ADAM metallopeptidase with thrombospondin type 1 motif 1 (*ADAMTS1*) and androgen receptor (*AR*) playing key roles in extracellular matrix remodeling and reproductive signaling, respectively (Supplementary Table S2). GO analysis of DEGs from the pairwise comparison between control and sham-inseminated SST at D30 showed enrichment of cell redox homeostasis and selenocompound metabolism (Supplementary Table S3).

When sham-inseminated and semen-inseminated SSTs were compared at D30 through a pairwise analysis, a total of 769 DEGs were identified (Supplementary Table S1). The top network constructed comprised of DEGs with functional roles in anti-apoptotic functions, fatty acid synthesis, and metabolism (Figure 4B). Increased anti-apoptotic gene expression, including tyrosine 3-monooxygenase/tryptophan 5-monooxygenase activation protein eta/gamma (*YWHAH*/*YWHAG*) and yes-associated protein 1 (*YAP1*) with predicted upstream regulation by class I phosphoinositide 3-kinases (PI3K class I), were seen in sham-inseminated samples. Gene expression in sham-inseminated samples was indicative of fatty acid synthesis, potentially driven by upstream regulator cytochrome-c oxidase, with ATP citrate lyase (*ACLY*) and fatty acid synthase (*FASN*) exhibiting increased expression in sham-inseminated samples. Within semen-inseminated samples, upregulation of succinyl-CoA:glutarate-CoA transferase (*SUGCT*), was identified, which plays a role in fatty acid metabolism. Both pairwise comparisons identified genes upregulated in sham-inseminated samples related to: 1) thyroid hormone metabolism and signaling from deiodinase 2 (*DIO2*) and thyroid hormone receptor beta (*THRB*); 2) cellular stress with ectodermal-neural cortex 1 (*ENC1*), growth arrest and DNA damage inducible gamma (*GADD45G*), coiled-coil-helix-coiled-coil-helix domain containing 4 (*CHCHD4*), and DDB1 and CUL4 associated factor 12 (*DCAF12*); and 3) cell cycle dynamics from tousled like kinase 1 (*TLK1*) and tubulin beta 4B class IVb (*TUBB4B*) as well as potential upstream regulation by cyclin-dependent protein kinase Cdk1/Cdc2 (*CDC2*). Upstream analysis also identified 312 potential regulators exhibiting significant activity, with 12 potential regulators exhibiting increased expression in sham-inseminated samples and 2 potential regulators exhibiting increased expression in semen-inseminated samples (Supplementary Table S2). GO analysis of DEGs from the pairwise comparison between sham-inseminated and semen-inseminated SST at D30 showed enrichment signal transduction, cellular response to fatty acid, ECM organization, and bleb assembly biological processes. In addition, glycosaminoglycan biosynthesis, calcium signaling, selenocompound metabolism, and ECM-receptor interaction KEGG pathways also showed enrichment (Supplementary Table S3).

**FIGURE 5:**
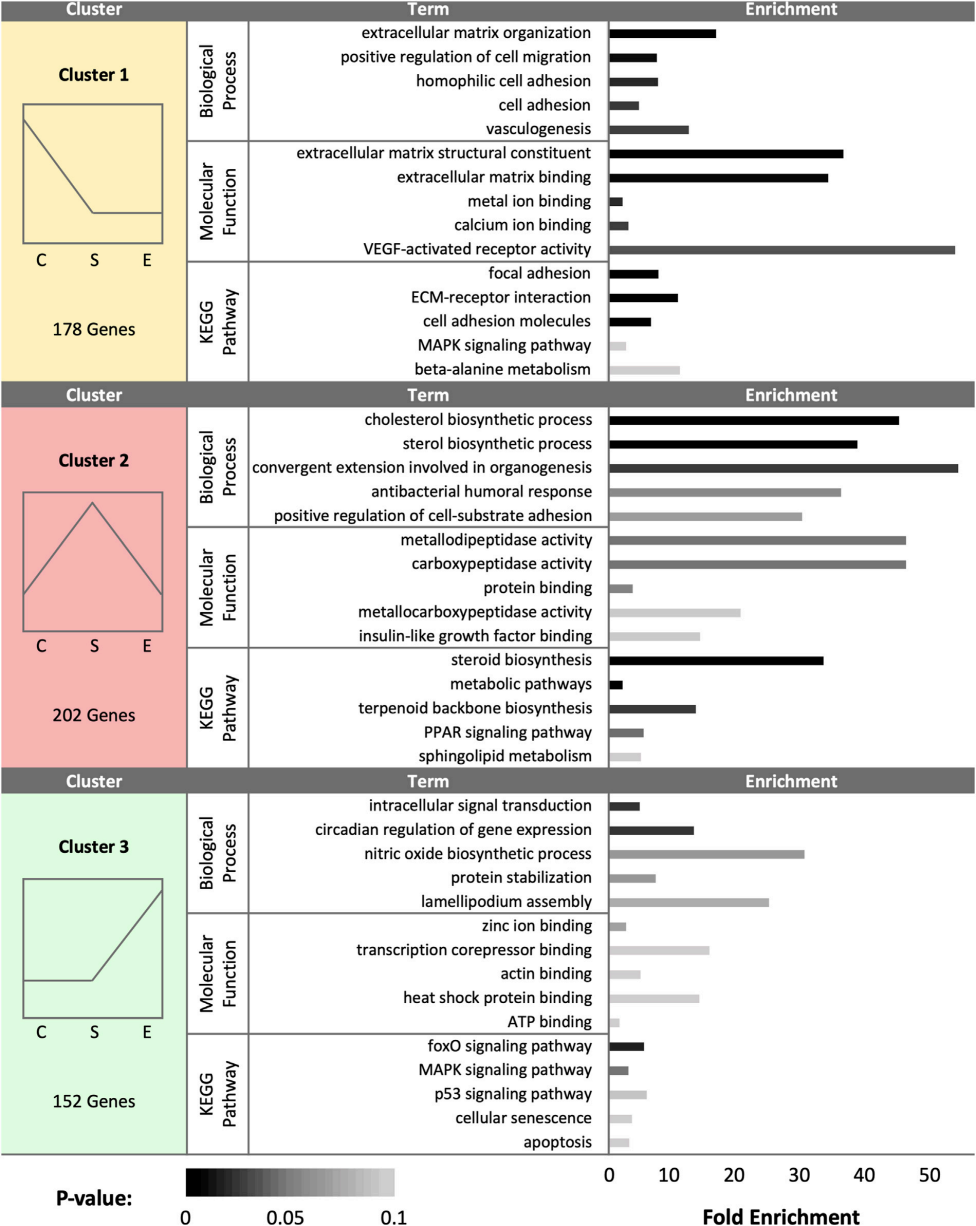
K-medoids cluster analysis of sperm storage tubules (SSTs) isolated from: control, sham-inseminated, and semen-inseminated at 90-days post-insemination (D90). The k-medoids cluster analysis tool from CLC Genomics (Qiagen, Valencia, CA) was used to identify genes exhibiting peak expression in treatment group. Genes included in the analysis were differentially expressed due to treatment (q < 0.05). Left panels show the expression profile and number of genes in each cluster (C = control, S = sham-insemination, E = semen-insemination). Right panels show enriched gene ontology (GO) term biological processes and molecular functions as well as KEGG pathway enrichment generated from Database for Annotation, Visualization, and Integrated Discovery (DAVID).

At day 90 (D90), a total of 532 DEGs were identified as statistically significant due to treatment group. Cluster analysis revealed a total of 178 DEGs upregulated in control samples, 202 DEGs upregulated in sham samples, and 152 DEGs upregulated in experimental samples (Figure 5). In control hens, enriched gene expression associated with ECM organization, cell adhesion and migration, vasculogenesis, and metal and calcium binding were identified. The pathway enrichment in control hen SSTs was identified as related to cell adhesion, ECM receptor interaction, MAPK signaling, and alanine metabolism. The sham-inseminated hen samples exhibited gene upregulation related to cholesterol and sterol biosynthesis, cell adhesion, peptidase activity, insulin-like growth factor binding, and humoral immune response. Pathway enrichment in sham-inseminated hen SSTs was identified as related to steroid biosynthesis, metabolic pathways, and PPAR signaling. Lastly, the semen-inseminated hen samples had increased expression levels for genes linked to nitric oxide biosynthesis, lamellipodium assembly, circadian gene regulation, and protein stabilization, along with zinc ion, corepressor, actin, heat shock protein, and ATP binding. Pathway enrichment in semen-inseminated hen SSTs was associate with foxO, MAPK, and p53 signaling as well as cellular senescence and apoptosis.

**FIGURE 6:**
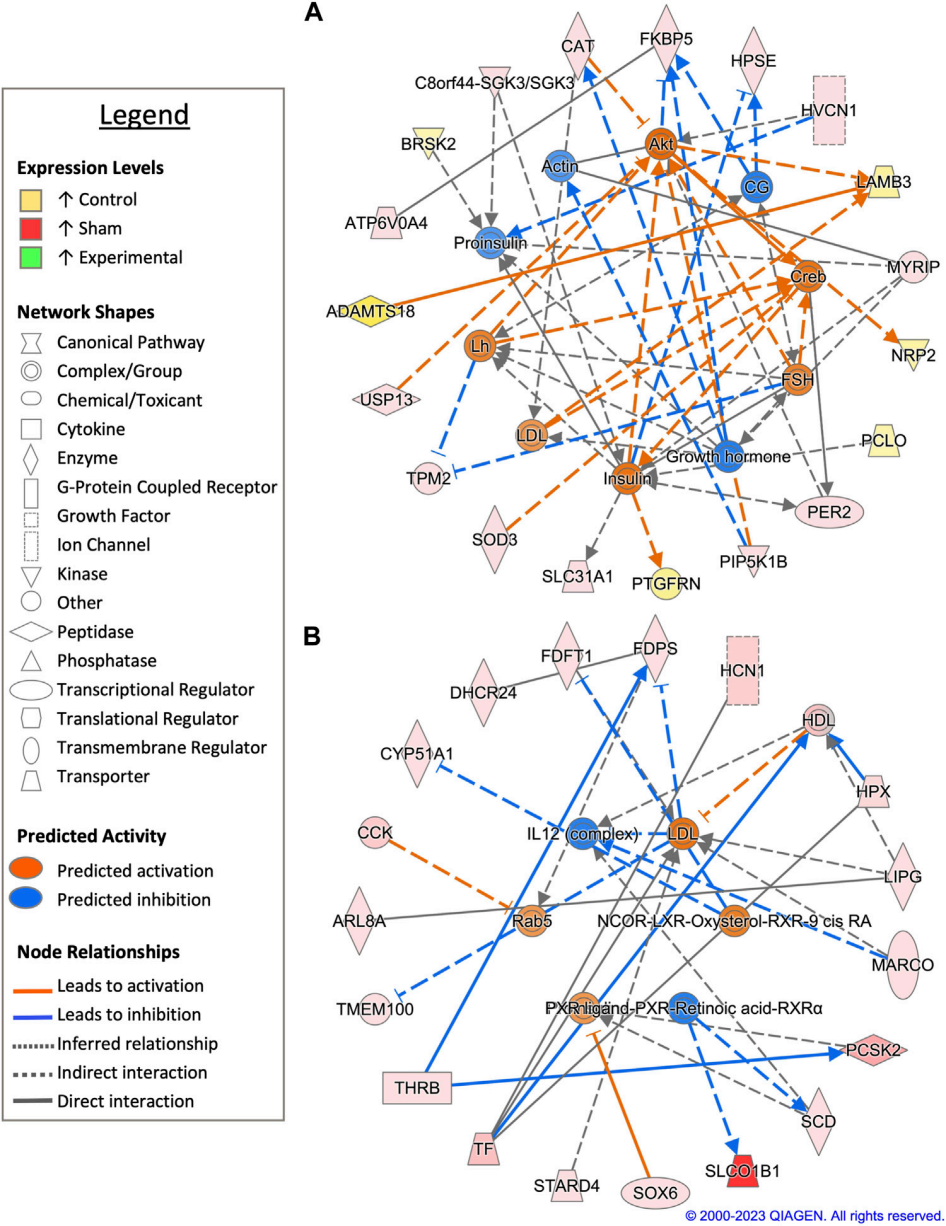
Network analysis of sperm storage tubules (SSTs) isolated at 90-days post insemination (D90). The core analysis tool from Ingenuity Pathway Analysis (IPA) (Qiagen, Valencia, CA) was used to biologically interpret differentially expressed genes (DEGs). Copyright permission from Qiagen has been obtained for use of the images presented. **(A)** The top network generated through pathway analysis of DEGs between control and sham-inseminated hens (FPKM > 1, *q*-value < 0.05, |fold change| > 1.5). **(B)** The top network generated through pathway analysis of DEGs between sham-inseminated and semen-inseminated hens (FPKM > 1, *q*-value < 0.05, |fold change| > 1.5).

Pairwise comparison between control and sham-inseminated SSTs at D90 generated a total of 118 DEGs, which were involved in extracellular matrix organization and insulin responsiveness (Figure 6A; Supplementary Table S1). A cytoskeletal upstream regulator, actin, was predicted to have increased expression along with cytoskeletal genes, heparanase (*HPSE*) and beta-tropomyosin (*TPM2*), in sham-inseminated samples. Conversely, in control samples, upregulation of basement membrane proteins and extracellular matrix degradation metalloproteinases, laminin subunit beta 3 (*LAMB3*) and ADAM metallopeptidase with thrombospondin type 1 motif 18 (*ADAMTS18*), was observed. Similar to the D30 timepoint, insulin activity was predicted to increase in control samples, with downstream targets such as BR serine/threonine kinase 2 (*BRSK2*) and prostaglandin F2 receptor inhibitor (*PTGFRN*) exhibiting increased expression levels in control samples. Upstream analysis identified 63 potential regulators exhibiting significant activity, with several regulators involved in immune function (NfkB-RelA, *PTPN13*, *ICOS*) (Supplementary Table S2). GO analysis of DEGs from the pairwise comparison between control and sham-inseminated SST at D90 showed enrichment of intracellular signal transduction and cell adhesion molecules (Supplementary Table S3).

In a pairwise comparison of sham-inseminated and semen-inseminated SSTs at D90, a total of 108 DEGs were identified (Supplementary Table S1). The top network identified in analysis was comprised of DEGs with roles in immune function, thyroid hormone signaling, and cholesterol metabolism (Figure 6B). The upstream immune regulator interleukin 12 (IL12), was predicted to have increased expression in sham-inseminated, along with increased expression of macrophage receptor with collagenous structure (*MARCO*). Thyroid hormone signaling genes, such as *THRB* and solute carrier organic anion transporter family member 1B1 (*SLCO1B1*) exhibited upregulation in sham-inseminated samples, like those at the D30 timepoint. Several genes with roles in cholesterol metabolism exhibited increased expression in the sham-inseminated samples, including cytochrome P450 family 51 subfamily A member 1 (*CYP51A1*), 24-dehydrocholesterol reductase (*DHCR24*), farnesyl-diphosphate farnesyltransferase 1 (*FDFT1*), farnesyl diphosphate synthase (*FDPS*), high density lipoprotein (*HDL*), and StAR related lipid transfer domain containing 4 (*STARD4*). Low density lipoprotein (LDL) was predicted to be activated in semen-inseminated samples. Like the D30 timepoint, both pairwise comparisons showed sham-inseminated samples had upregulation of genes related to cellular stress, namely, catalase (*CAT*), superoxide dismutase 3 (*SOD3*), and hemopexin (*HPX*). Upstream analysis identified 129 potential regulators exhibiting significant activity, with several regulators involved in fatty acid metabolism (*ELOVL3*, *SCD*), glycolysis/gluconeogenesis (*ACSS2*, *PGK1*), and insulin signaling (*HRAS*, *INSR*, *RPTOR, SREBF1*) (Supplementary Table S2). GO analysis of DEGs from the pairwise comparison between sham-inseminated and semen-inseminated SST at D90 showed enrichment of lipid metabolism and steroid biosynthesis (Supplementary Table S3).

**FIGURE 7:**
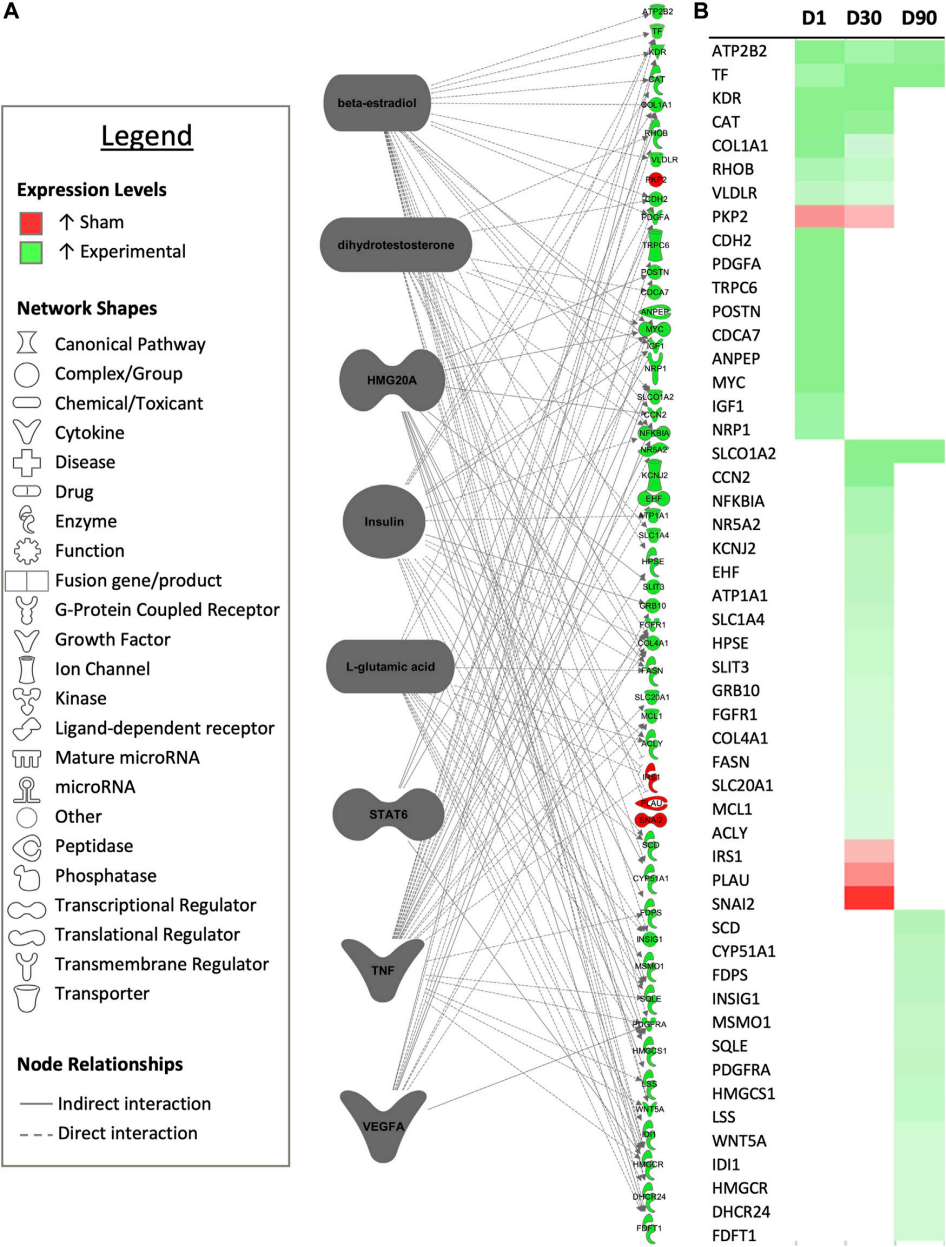
Common upstream regulators occurring multiple timepoints. **(A)** Common predicted upstream regulators for each experimental timepoint from the core analysis tool of Ingenuity Pathway Analysis (Qiagen, Valencia, CA) are presented. Only upstream regulators with significant predicted activity (|z-score| > 2, *p* < 0.05) are represented. Downstream targets exhibiting differential expression between sham-inseminated and semen-inseminated samples are presented (FPKM > 1, *q*-value < 0.05, |fold change| > 1.5). **(B)** Heat map expression profiles for each target gene 1-day (D1), 30-days (D30), and 90-days (D90) post-insemination. Green represents increased expression in semen-inseminated samples, while red represents higher expression in sham-inseminated samples.

Upstream analysis revealed 10 predicted upstream regulators based upon DEGs between sham-inseminated and semen-inseminated pairwise comparisons that were common to more than one timepoint, with one of the predicted upstream regulators, insulin, also appearing in pairwise comparisons between control and sham inseminated samples as well. Dihydrotestosterone (*DHT*), signal transducer and activator of transcription 6 (*STAT6*), tumor necrosis factor (*TNF*), and vascular endothelial growth factor A (*VEGFA*) were identified as upstream regulators at the D1 and D30 timepoints, while beta-estradiol, high mobility group 20A (*HMG20A*), insulin, and L-glutamic acid were identified as upstream regulators at the D30 and D90 timepoints. In total, these regulators had 60 downstream targets that exhibited differential expression between sham-inseminated and semen-inseminated samples, with considerable target overlap between regulators (Figure 7). Downstream targets were associated with the following biological pathways: steroid biosynthesis, calcium signaling, cholesterol biosynthesis, fatty acid biosynthesis, cell adhesion, fibroblast and endothelial cell proliferation, plasminogen activation, angiogenesis, insulin-like and vascular endothelial growth factor receptor signaling, and extracellular matrix organization (Supplementary Figure S2).

RT-qPCR was performed on six randomly selected genes using all treatment groups, timepoints, and replicates to assess the reliability of results obtained through RNA sequencing. For each gene analyzed, the log_2_ fold change obtained through RT-qPCR showed strong correlation with the log_2_ fold change obtained through RNA sequencing (Supplementary Figures S3), with the R_2_ ranging from 0.7992 for *KCNMB1* to 0.8867 for *SPARC* and an average R_2_ of 0.85 for the six genes examined.

## 4 Discussion

Previous analysis of the SST transcriptome demonstrated temporal changes throughout the duration of fertility (Brady et al., 2022). In the current study, it has now been established that the SST transcriptome is also impacted by the presence of semen and the act of insemination. Between the treatment groups, comparisons between control and sham-inseminated hens yielded fewer DEGs, while more stark differences were observed in comparisons between sham-inseminated and semen-inseminated hens. Transcriptome differences, particularly in the semen-inseminated group, were measurable for up to 90 days post-insemination. Analysis of the DEGs acquired through analysis revealed upregulation of pathways relating to classical and non-classical reproductive signaling, cytoskeletal remodeling, physiological parameters of the local UVJ environment, and cellular metabolism in semen-inseminated samples. In contrast, sham-inseminated samples were observed to have upregulation of immune pathways and non-reproductive endocrine hormones.

Classical reproductive signaling was noted in each comparison across the three timepoints. It has been previously established that SST function is regulated by steroid hormones, such as progesterone and estradiol, that target sperm-release and sperm storage duration (Ito et al., 2011; Yang et al., 2020). Less is known about direct gonadotropin stimulation on SST function. In the Japanese quail, receptors for LH, FSH, progesterone, and estradiol have been detected in the UVJ, with expression levels increasing leading up to the initiation of egg lay (Khillare et al., 2018). Expression of LH and FSH receptors, coupled with the predicted upstream regulation of LH and FSH in the current dataset, indicates that gonadotropins may act directly on SST, in addition to acting indirectly through stimulation of follicular steroid hormone production. In the analysis, LH and FSH were predicted to be activated at D1 in semen-inseminated samples, but not predicted to be active until D30 and D90 timepoints in control hens. Within the cluster analysis at D1, GO term and KEGG pathway enrichment included hormone-mediated signaling pathway and activation of adenylate cyclase activity, both related to *FSHR* and *LHCGR* expression, in the sham-inseminated group. These D1 pathway results indicate that the act of insemination, rather than the presence of semen, can modulate SST gonadotropin responsiveness. Sustained gonadotropin responsiveness is evident at the D30 timepoint in semen-inseminated hens, with GO term and KEGG pathway enrichment including GnRH signaling and steroid hormone biosynthesis. Within the pairwise comparisons at each timepoint, upstream regulators related to steroid hormone signaling, such as estrogen receptors 1 and 2 (*ESR1* and *ESR2*), beta-estradiol, progesterone receptor (*PGR*), and progesterone, were predicted to have significant activity in the comparisons between sham-inseminated and semen-inseminated samples. In addition to direct action of gonadotropins on SST function, the predicted upstream regulators also indicate indirect regulation of gonadotropins on SST function through steroid hormone signaling.

Non-classical aspects of reproductive signaling were also observed, including androgen regulation and calcium signaling. Increased circulating androgens have been associated with hibernation related sperm storage in turtles and with sperm storage in bats (Roy and Krishna, 2010; Liu et al., 2016). In avian species, androgen derivatives have been identified in the serum metabolome of hens with increased sperm storage capacities (Yang et al., 2021). In the current dataset, dihydrotestosterone was identified as a potential upstream regulator common to both the D1 and D30 timepoints, indicating that androgen regulation may be responsible for downstream gene expression differences seen between the sham-inseminated and semen-inseminated hens. Upstream analysis through pairwise comparisons of sham-inseminated and semen-inseminated samples also predicted androgen receptor (AR) and testosterone to have significant activity, further supporting the role of androgens in SST function. Additionally, calcium signaling and ion binding appeared in the enrichment analysis of control and sham-inseminated samples at each timepoint. Calcium stimulates sperm motility and respiration (Ashizawa et al., 1992) *in vitro;* however, with calcium signaling and ion binding enrichment not seen in semen-inseminated samples, one could speculate that calcium regulation may be required for prolonged sperm storage. On the other hand, calcium ions have been visualized in SSTs with co-localization with sperm cells (Riou et al., 2017). Calcium involvement in sperm storage is further complicated by vast calcium mobilization to the oviduct for egg formation. Further studies are necessary to examine the role of both androgens and calcium in SST function.

Apart from reproductive hormones, other endocrine hormones were identified as upstream regulators, specifically thyroid hormone and insulin. Within the sham-inseminated treatment, upregulation of thyroid hormone receptors, deiodinases, and transporters was observed at multiple timepoints. The role of thyroid hormone regulation on egg lay initiation and cessation has been well characterized; but recently, the impact of thyroid hormone treatment on broiler breeder UVJ gene expression has been distinguished. Thyroid hormone treatment decreased expression of carbonic anhydrase, avidin-related protein 2, and TGF beta, which play key roles in SST function (Hatami et al., 2018; Saemi et al., 2018). Upregulation of thyroid hormone related genes in the sham-inseminated hens may serve to downregulate key SST functions when sperm are not present. The alteration of circulating thyroid hormones in chickens through inhibition of thyroid hormone synthesis is reported to increase fertility rates (Marks, 1969; Elnagar et al., 2005). Downregulation of thyroid hormone receptors, deiodinases, and transporters in the semen-inseminated group compared with the sham-inseminated group suggest that the presence of semen may modulate SST receptiveness to circulating thyroid hormones. Insulin was found as a predicted upstream regulator in the pairwise comparisons made between control and sham-inseminated hens at each timepoint. In addition, insulin receptor repressor, *SPRY4* exhibited increased expression in semen-inseminated samples at D1. Circulating insulin levels have been associated with decreased fertility levels (Chen et al., 2006) and insulin receptor expression was found to increase with age in chickens, correlating to decreased fertility levels (Yang et al., 2021). Circulating insulin levels are regulated, in part by estradiol levels, with estradiol decreasing insulin levels (Jaccoby et al., 1995). With estradiol identified as a possible upstream regulator of semen-inseminated gene expression, increased estradiol levels could also decrease circulating insulin and/or insulin signaling in the semen-inseminated hens.

Formation and maintenance of SSTs throughout the laying cycle involves cytoskeletal remodeling and extracellular matrix organization, including actin microfilament formation and chondrocyte proliferation (Mendonca et al., 2019; Nabil et al., 2022). Cytoskeletal remodeling and extracellular matrix organization regulate other physiological processes, such as angiogenesis, that are important for nutrient delivery. Cluster analysis identified numerous GO terms and KEGG pathways associated with cytoskeletal remodeling and extracellular matrix organization were identified in all three treatment groups at the D30 and D90 timepoints. However, these terms are enriched only in the semen-inseminated group at D1. Further, pairwise comparisons of sham-inseminated and semen-inseminated samples resulted in DEGs with enrichment in angiogenesis and fibroblast proliferation biological processes at D1. Upregulation of cytoskeletal genes was also previously identified in semen-inseminated SSTs compared to sham-inseminated samples (Long et al., 2003). Within the turkey industry, pre-lay inseminations are utilized to increase SST filling rates and increase early fertility rate (Brillard and Bakst, 1990). Results from this study suggest that the presence of semen accelerates SST development at this early timepoint and could explain why pre-lay insemination strategies lead to improved SST function. Signaling of Wnt has been previously implicated in SST structure in poultry (Yang et al., 2023), and in this analysis, Wnt signaling shows extensive enrichment in semen-inseminated samples at the D30 timepoint.

The local UVJ environment is dictated by nutrient availability and transport, oxidative status, and pH. In this analysis, *HMG20A* and *VEGFA* were identified as potential upstream regulators in multiple comparisons between sham-inseminated and semen-inseminated samples (*HMG20A* at D30/D90 and *VEGFA* at D1/D30), with vascular endothelial growth factor binding highly enriched in semen-inseminated samples at D1. The *HMG20A* gene regulates epithelial cell characteristics, while *VEGFA* is a key regulator of angiogenesis (Silva and Mooney, 2010; Rivero et al., 2015). Both epithelial cell function and angiogenesis could have profound effects on nutrient availability the local SST environment, ultimately impacting sperm storage capabilities. Ion transport of calcium, iron, potassium, sodium, zinc, and selenium was enriched across both treatments and timepoints. In particular, transferrin, an iron transporter, was upregulated in the semen-inseminated samples at the D1 timepoint and has been shown to be associated with sperm storage in poultry (Matsuzaki et al., 2020; Kubota et al., 2023). In addition to nutrient availability, the local SST environment is also impacted by pH and reactive oxygen species (ROS). The carbonic anhydrase activity in SSTs has been associated with pH maintenance, and multiple carbonic anhydrases were identified as upregulated in the semen-inseminated group (Han et al., 2019). Oxidative stress generates ROS, leading to cellular and DNA damage and several oxidative stress genes (*CAT, HPX, ENC1*) exhibited upregulation in the sham-insemination group across the different timepoints. The presence of ROS in tissue that store sperm is associated with decreased sperm quality and fertility potential in chickens (Kheawkanha et al., 2023), with selenium supplementation mitigating the ROS damage (Chauychu-Noo et al., 2021). Additional work is required to determine critical components of the local UVJ environment that are necessary for prolonged sperm storage success.

Lastly, immune response is directly related to sperm storage capabilities. It has been previously established that mating and artificial insemination upregulate immune related genes in SSTs (Atikuzzaman et al., 2015; Brady et al., 2022). In this analysis, upregulation of immune related genes as well as predicted upstream regulators with immune functions was seen in the sham-inseminated samples compared to control samples. Pairwise comparisons between control and sham-inseminated samples identified several immune related upstream regulators at D1 (*NOD2*, DUB, caspase), D30 (*CHUK*, *CXCR5*, *IL10RB*, *TRIM32*), and D90 (NfkB-RelA, *PTPN13*, *ICOS*) timepoints, indicating that the immune response is sustained to some degree for an extended period. When sham-inseminated and semen-inseminated samples were compared, sham-inseminated samples still exhibited higher expression of immune related genes and regulators. This could be indicative that the presence of semen in the SST may reduce the localized oviduct immune response to increase or preserve semen viability within the avian reproductive tract. Heightened immune responses were also seen in subfertile chicken oviduct fluid, with increased immunoglobulin receptor proteins, resulting in greater immune responsiveness (Riou et al., 2019). Additionally, two of the predicted upstream regulators identified in multiple comparisons, STAT6 and TNF, are known regulators of immune function (Kowssar et al., 2013; Rohde et al., 2018). Further research is needed to elucidate the different types of immune response to the act of insemination and to the presence of sperm.

From this study, the presence of semen 1) increased classical and non-classical reproductive signaling, 2) accelerated cytoskeletal remodeling, 3) buffered physiological parameters of the local UVJ environment, and 4) upregulated cellular metabolism. Conversely, the act of insemination 1) upregulated immune pathways and 2) increased non-reproductive endocrine hormones, such as thyroid hormone and insulin. Additional research is needed to further validate the upstream regulators predicted through this study and determine the role of these regulators on SST functionality. This study has laid the groundwork to characterize the transcriptome changes due to artificial insemination that will potentially aid in optimization in turkey hen insemination and *in vitro* semen storage protocols.

## Data Availability

The datasets presented in this study can be found in online repositories. The names of the repository/repositories and accession number(s) can be found below: https://www.ncbi.nlm.nih.gov/bioproject, PRJNA1022824 https://www.ncbi.nlm.nih.gov/, Biosamples: SAMN37628532- SAMN37628544, SAMN37628829- SAMN37628838, SAMN37629530- SAMN37629533.

## Supplementary material

SUPPLEMENTARY FIGURE S1 Mapping and fragment statistics of control (C), sham-inseminated (S), and semen-inseminated (E) sperm storage tubules (SST) at 1-day (D1), 30-days (D30), and 90-days (D90) post-insemination. **(A)** Raw reads obtained for each sample. **(B)** Percentage of reads mapped in pairs, reads mapped in broken pairs, and reads not mapped.

SUPPLEMENTARY FIGURE S2 Functional annotation analysis of target genes of upstream regulators common to more than one pairwise comparison. Enriched gene ontology (GO) term biological processes and molecular functions as well as KEGG pathway enrichment generated from Database for Annotation, Visualization, and Integrated Discovery (DAVID) for target genes of the upstream regulators common to more than one pairwise comparison.

SUPPLEMENTARY FIGURE S3 Confirmation by RT-qPCR of gene expression results obtained through RNA sequencing. Pearson’s correlation analysis of log2 fold changes obtained through RNA sequencing and RT-qPCR for **(A)** cytoglobin (CYGB), **(B)** serine peptidase inhibitor, kunitz type 4 (SPINT4), **(C)** potassium calcium-activated channel subfamily M regulatory beta subunit 1 (KCNMB1), **(D)** serpin family B member 5 (SERPINB5), **(E)** secreted protein acidic and cysteine rich (SPARC), and **(F)** stearoyl-CoA desaturase (SCD) in control **(C)**, sham-inseminated (S), and semen-inseminated **(E)** sperm storage tubules (SST) at 1-day (D1), 30-days (D30), and 90-days (D90) post-insemination.

SUPPLEMENTARY TABLE S1 Differential expression output obtained from CLC Genomics Workbench for each pairwise comparison. Differentially expressed genes (DEGs) are highlighted in yellow and meet the following conditions: q < 0.05, |fold change|>1.5, and FPKM>1.

SUPPLEMENTARY TABLE S2 Upstream analysis output for each pairwise comparison obtained from Ingenuity Pathway Analysis. Upstream regulators listed meet the following conditions: |z-score|>2 and *p* < 0.05.

SUPPLEMENTARY TABLE S3 Gene ontology (GO) and KEGG pathway analysis for each pairwise comparison. Listed terms and pathways meet the following conditions: gene count>2 and *p* < 0.05.
