# Supplementary figures and images for: Turkey hen sperm storage tubule transcriptome response to artificial insemination and the presence of semen
Source: Front Physiol. 2024 Jan 8;14:1305168. doi: 10.3389/fphys.2023.1305168 (PMC10801083; doi:10.3389/fphys.2023.1305168)

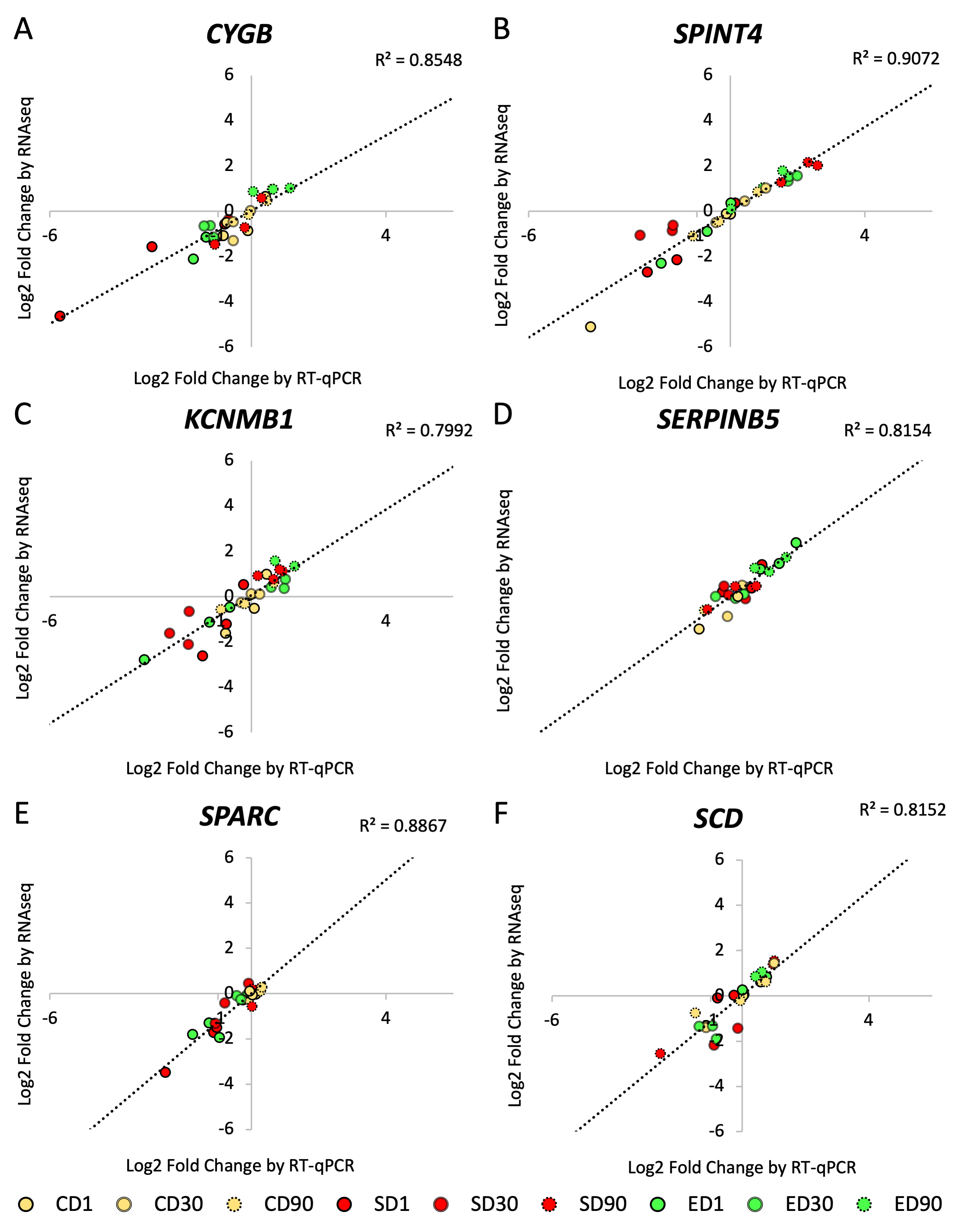

Supplement: Supplementary file 1 [file Image3.TIFF]

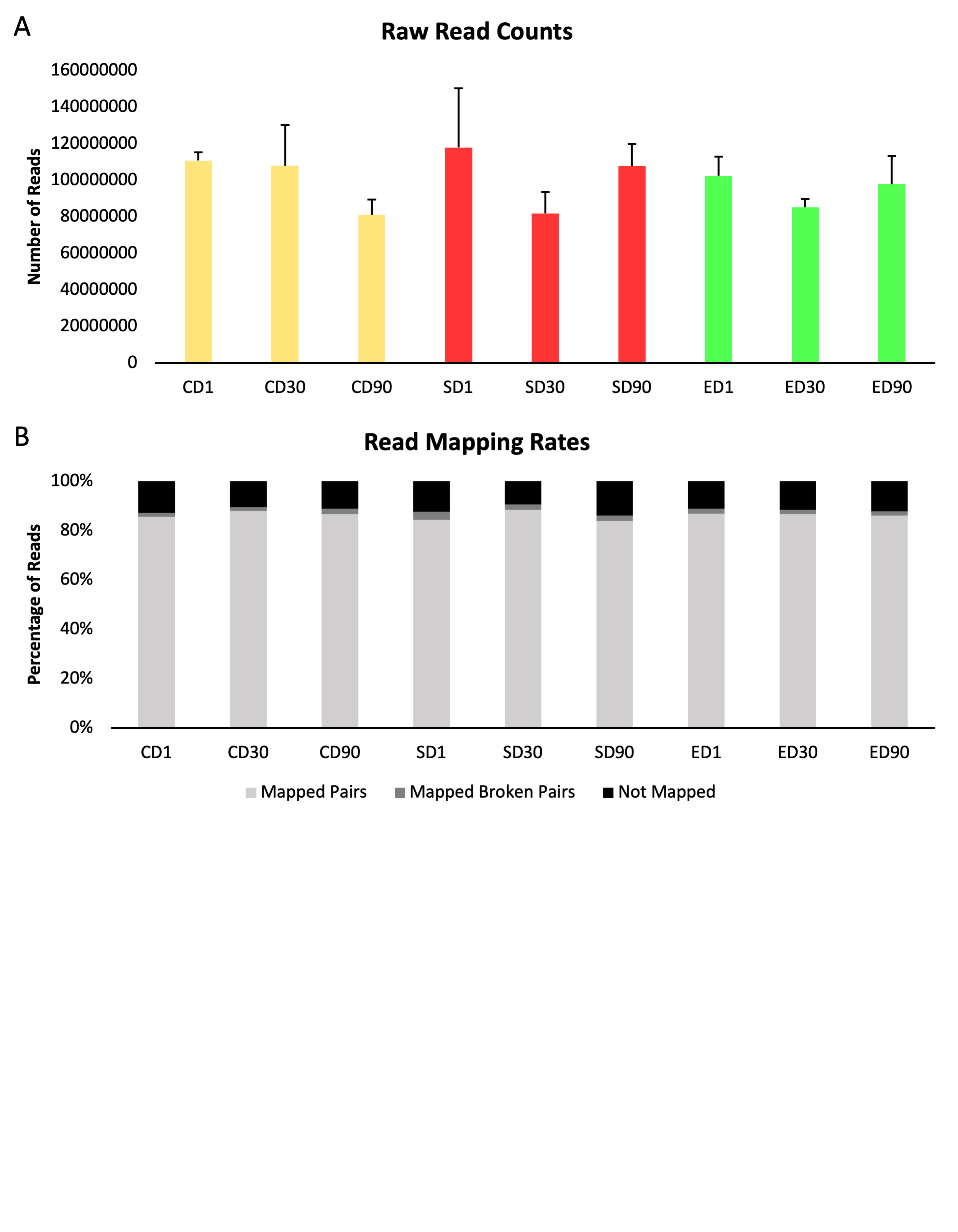

Supplement: Supplementary file 3 [file Image1.TIFF]

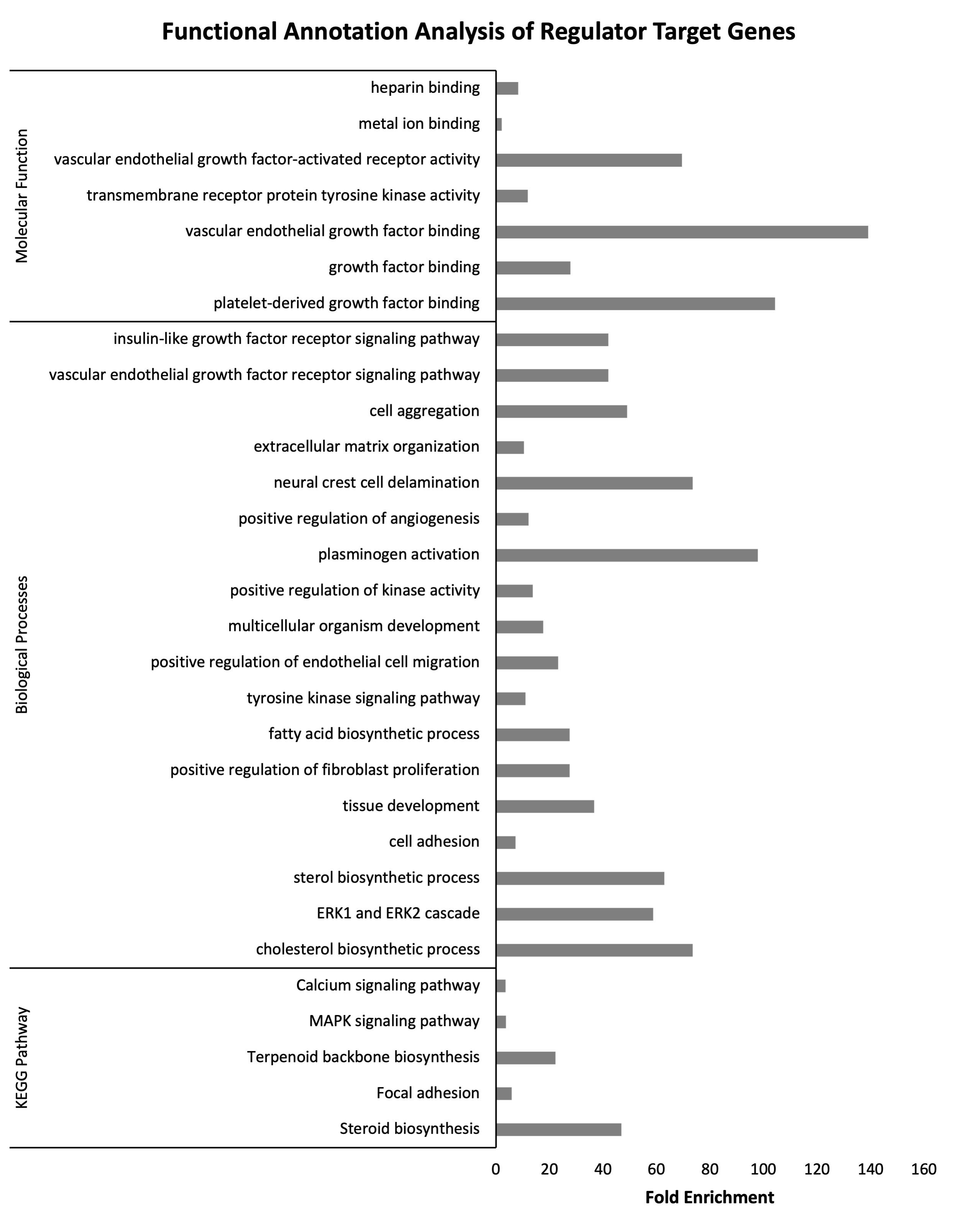

Supplement: Supplementary file 6 [file Image2.TIFF]
